# The effects of pharmacological accommodation and cycloplegia on axial length and choroidal thickness

**DOI:** 10.5935/0004-2749.20210015

**Published:** 2025-02-02

**Authors:** Alperen Bahar, Gökhan Pekel

**Affiliations:** 1 Ophthalmology Department, Pamukkale University, Denizli, Turkey

**Keywords:** Corneal pachymetry, Choroid, Corneal topography, Axial length, eye, Mydriatics/pharmacology, Pilocarpine/pharmacology, Accommodation, ocular, Paquimetria corneana, Coroide, Topografia da córnea, Comprimento axial do olho, Midriáticos/farmacologia, Pi locarpina/farmacologia, Acomodação ocular

## Abstract

**Purpose:**

To investigate the effects of pharmacological accommodation and cycloplegia on ocular measurements.

**Methods:**

Thirty-three healthy subjects [mean (±SD) age, 32.97 (±5.21) years] volunteered to participate in the study. Measurement of the axial length, macular and choroidal thickness, refractive error, and corneal topography, as well as anterior segment imaging, were performed. After these pro cedures, pharmacological accommodation was induced by applying pilocarpine eye drops (pilocarpine hydrochloride 2%), and the measurements were repeated. The measurements were repeated again after full cycloplegia was induced using cyclopentolate eye drops (cyclopentolate hydrochloride 1%). The correlations between the measurements were evaluated.

**Results:**

A significant increase in subfoveal choroidal thickness after applying 2% pilocarpine was identified (without the drops, 319.36 ± 90.08 µm; with pilocarpine instillation, 341.60 ± 99.19 µm; with cyclopentolate instillation, 318.36 ± 103.0 µm; p<0.001). A significant increase in the axial length was also detected (without the drops, 23.26 ± 0.83 mm; with pilocarpine instillation, 23.29 ± 0.84 mm; with cyclopentolate instillation, 23.27 ± 0.84 mm; p=0.003). Comparing pharmacological accommodation and cycloplegia revealed a significant difference in central macular thickness (with pilocarpine instillation, 262.27 ± 19.34 µm; with cyclopentolate instillation, 265.93 ± 17.91 µm; p=0.016). Pilocarpine-related miosis (p<0.001) and myopic shift (p<0.001) were more severe in blue eyes vs. brown eyes.

**Conclusion:**

Pharmacological accommodation may change ocular measurements, such as choroidal thickness and axial length. This condition should be considered when performing ocular measurements, such as intraocular lens power calculations.

## Abstract (pt)

**Objetivo:**

Investigar os efeitos da acomodação farmacológica e da cicloplegia nas medições oculares.

**Métodos:**

participaram do estudo 33 voluntários saudáveis (média de idade [± DP], 32,97 anos [± 5,21 anos]). Foram medidos o comprimento axial, a espessura macular e coroidal e o erro refrativo, bem como realizados exames de imagem da topografia corneana e do segmento anterior. Em seguida, foi induzida a acomodação farmacológica aplicando-se colírio de pilocarpina (cloridrato de pilocarpina a 2%) e as medições foram repetidas nos participantes. As mesmas medições foram repetidas depois de induzir a cicloplegia completa com colírio de ciclopentolato (cloridrato de ciclopentolato a 1%) e foram avaliadas as correlações entre as medidas.

**Resultados:**

Identificou-se aumento significativo da espessura coroidal subfoveal com o uso da pilocarpina a 2% (sem colírio, 319,36 ± 90,08 µm; com a instilação de pilocarpina, 341,60 ± 99,19 µm; com a instilação de ciclopentolato, 318,36 ± 103,0 µm; p<0,001). Detectou-se também aumento significativo do comprimento axial (sem colírio, 23,26 ± 0,83 mm; com a instilação de pilocarpina, 23,29 ± 0,84 mm; com a instilação de ciclopentolato, 23,27 ± 0,84 mm; p=0,003). Ao se comparar a acomodação farmacológica e a cicloplegia, houve diferença significativa na espessura macular central (com a instilação de pilocarpina, 262,27 ± 19,34 µm; com a instilação de ciclopentolato, 265,93 ± 17,91 µm; p=0,016). Observou-se que a miose associada à pilocarpina (p<0,001) e o desvio miópico (p<0,001) foram mais severos nos olhos azuis que nos castanhos.

**Conclusão:**

A acomodação farmacológica pode alterar medidas oculares como a espessura da coroide e o comprimento axial. Essa possibilidade deve ser levada em consideração ao se efetuarem medições oculares, tais como cálculos de potência de lentes intraoculares.

## INTRODUCTION

At ophthalmology clinics, various measurements are taken routinely for diagnostic purposes, using various examinations, such as autorefraction, ocular coherence tomography, corneal topography, and ocular biometry. Patients are either diagnosed or their treatments are updated based on these measurements, which may be affected by patient-related or environmental factors. This study was performed to assess the effect of cycloplegia and pharmacological accommodation on ocular measurements.

Previous studies reported controversial findings regarding the effects of accommodation and cycloplegia on ocular measurements. Although some studies reported no changes in choroidal thickness after cycloplegia, most found significant changes^(1-4)^. Several reported that measurements, such as corneal curvature, anterior chamber depth, and axial length, are affected after phar macological accommodation and cycloplegia^(5-9)^. Although various studies have revealed measurements are affected in cycloplegic eyes, studies on accommodative eyes have been limited. Furthermore, to our knowledge, no study has compared cycloplegia with accommodation.

In this study, we aimed to investigate differences in ocular measurements when the patients are in normal, cycloplegic, and accommodative conditions. With these examinations, we hope to contribute to the standardization of ocular measurements and advance ocular biophysical knowledge. In addition, the increased sensitivity to pilocarpine among participants with blue eyes, which was noticed during the study, indicated that eye color should be included in the study.

## METHODS

Our study included 36 adult participants who were staff at our university hospital. All study participants provided signed informed consent after the nature and possible consequences of the study were explained to them at the time of study enrollment. Three participants were excluded because, based on test results, they did not meet the study inclusion criteria. The 33 individuals that participated included 15 men and 18 women. Only participants’ right eyes were studied. The study was conducted in accordance with the Helsinki Declaration and approved by the ethics committee. The inclusion criteria for research were as follows:

1. Age: 20-40 years.
2. Visual acuity: 20/20 with Snellen chart.
3. Normal anterior and posterior segment examination findings in ophthalmological examination.
4. No diagnosis of glaucoma or uveitis.
5. No systemic diseases.
6. RefracStion error within limits of ±4.00 diopters

The measurements were performed at noon when the participants were satiated. The first measurements were acquired without applying drops. Subsequently, within a 5 min interval, three drops of pilocarpine (2% pilocarpine hydrochloride, Pilosed, Turkey) were administered to participants’ eyes, and the measurements were performed again 30 min later. After one week of washout, the measurements were repeated for each participant prior to, and 45 min after, applying the cyclopentolate drops (within a 5 min interval, three drops) (1% cyclopentolate hydrochloride, Sikloplejin, Turkey). If the pupils were reactive to light, additional drops were administered until no reaction to light was observed before measurements were taken. The dose of the drops and the application period were selected in accordance with similar studies^(5,10)^. The measurements performed before the drops were compared, and no significant difference was found. Therefore, the first measurements were taken as data in statistical comparison.

The following measurements were obtained for participants, in this order:

1. Corneal topography; Pentacam^®^HR (Oculus, Wetzlar, Germany).
2. Macular, optical disk, and enhanced depth ima-ging optical coherence tomography; Spectralis^®^ (Heidelbergengineering, Heidelberg, Germany).
3. Axial length; NIDEK Optical Biometer AL-Scan (NIDEK CO. LTD, Gamagori, Japan).
4. Refraction and pneumatic intraocular pressure (mmHg); NIDEK TONOREF™ II, (NIDEK CO., LTD, Ga magori, Japan).
5. 5Uncorrected and corrected visual acuity (using the Snellen chart).
6. Front segment photography; DC-4 imaging system (Topcon Corporation, Tokyo, Japan).

All measurements were carried out by the same expert technician. During OCT, imaging was performed with subjects’ foveas placed at the center; for other examinations, the patients were asked to focus on the light source. Topography assessment was performed using all Scheimpflug images that had the desired clarity and fully appropriate image quality. Choroidal imaging was performed by a single-line scan using an optical coherence spectrometer (Spectralis^®^, Heidelbergengineering, Heidelberg, Germany) in the EDI mode. During scanning, the number of repetitive image acquisitions of the same cross-section was set to 100, and the same cross-section scans could be acquired using an eye-tracking program. Choroidal thickness was set as the distance between the outer edge of the hyper-reflective retinal pigment epithelium and the inner edge of the sclera in the subfoveal region. It was measured manually using onboard software (Heidel Berggeye Explorer 1.7.0.0). Each measurement was repeated three times, and the median value was selected. The participants’ eye color was classified into four-blue, green, hazel, and brown-after they were screened through the Topcon DC-4 imaging system.

The data obtained were coded and transferred into the computer program. The Windows Statistical Package for Social Sciences (SPSS Inc.) 21.0 software was used for statistical evaluation. The sample size was chosen using the following settings: alpha: 0.05; beta: 0.20; and standard effect size: 0.70, in *t*-test table. Descriptive findings were expressed as the mean ± standard deviation. The data normality was examined using the Kolmogorov-Smirnov test. One-way ANOVA for multiple comparisons and independent samples *t*-test for binary comparisons were used among the groups. Pearson’s correlation test was used for correlation analysis. The Mann-Whitney *U* test was used for the comparison of brown and blue eyes.

## RESULTS

Among the 33 volunteers who participated in the study, 15 were men and 18 were women. The mean age was 32.94 ± 5.2 years (21-40 years), and the colors of their irises were as follows: blue, 8 participants (24.2%); green, 1 (3%); hazel, 4 (12.1%); and brown, 20 (60.6%). The mean axial length was 23.26 ± 0.8 mm (22.14-25.50 mm) and the mean spherical refraction error rate was -0.49 ± 0.87 D (Table 1).

**Table 1:** Eye measurements performed without the use of drops

Measurements (W/O Drops) (n=33)	Minimum	Maximum	Mean	SD
Age	21	40	32.9	5.2
Spherical refraction (D)	-2.00	1.25	-0.49	0.87
Cylindrical refraction (D)	-2.75	0.0	-0.66	0.62
Axial Length (mm)	22.14	25.50	23.26	0.83
Subfoveal choroidal thickness (µm)	183	509	319.36	90.08
Central corneal thickness (µm)	442	578	537.54	30.72
Iridocorneal angle	25.10	51.10	35.10	6.36
Anterior segment volume (µL)	100	220	159.57	34.99
Anterior segment depth (mm)	2.19	3.43	2.85	0.32

### Subfoveal choroidal thickness correlation analysis

The participants’ mean subfoveal choroidal thickness was 319 ± 90.08 µm. The difference in subfoveal choroidal thickness between men and women was not statistically significant (p=0.35). There was no significant correlation between subfoveal choroidal thickness measured without drops and the ocular parameters [axial length (*r*=-0.061, p=0.76), intraocular pressure (*r*=0.11, p=0.54), central corneal thickness (*r*=0.047, p=0.79), and anterior segment volume (*r*=-0.128, p=0.47)].

### Comparison of measurements performed without drops and with pilocarpine and cyclopentolate drops

There was a significant increase in choroidal thickness after pharmacological accommodation with pilocarpine (p<0.001), as well as a significant increase in the axial length (p=0.003).

The mean central macular thickness, measured without the use of drops, was 264.06 ± 19.78 µm. It was 262.27 ± 19.34 µm in participants who received pilocarpine instillation and 265.70 ± 17.91 µm in those who underwent cyclopentolate instillation (p=0.013). A binary analysis showed that the difference was caused by the difference in the measurements with pilocarpine and cyclopentolate (p=0.016). There was no difference between the cyclopentolate-without drops and pilocarpine-without drops measurements (p=0.487).

No significant differences were observed among the groups in terms of central corneal thickness, iridocorneal angle, flat K (K1), steep K (K2), and maximum K values (Kmax) (p>0.05 for all). The anterior segment depth (mm) anterior segment volume (µL) values were significantly higher after cyclopentolate instillation and lower after pilocarpine instillation (Table 2).

**Table 2:** Comparison of the measurements performed in eyes without the use of drops and in accommodated and cycloplegic eyes. Significant differences are shown in bold

Measurements	W/O Drops (Mean ± SD) (n=33)	Pilocarpine hydrochloride 2% (Mean ± SD) (n=33)	Cyclopentolate hydrochloride 1% (Mean ± SD) (n=33)	P^a^ value
Central corneal thickness (µm)	537.54 ± 30.72	538.00 ± 31.42	540.12 ± 33.55	0.093
Iridocorneal angle	35.10 ± 6.36	35.53 ± 4.84	34.36 ± 6.94	0.62
Anterior segment depth (mm)	2.85 ± 0.32	2.77 ± 0.37	2.97 ± 0.29	<0.001
Anterior segment volume (µL)	159.57 ± 34.99	141.90 ± 27.91	170.16 ± 32.69	<0.001
K_1_ (D)	42.72 ± 1.50	42.73 ± 1.52	42.70 ± 1.59	0.47
K_2_ (D)	43.67 ± 1.54	43.67 ± 1.54	43.67 ± 1.57	0.98
K max (D)	43.19 ± 1.51	43.18 ± 1.51	43.17 ± 1.58	0.95
Central macular thickness(µm)	264.06 ± 19.78	262.27 ± 19.34	265.93 ± 17.91	0.013
Subfoveal choroidal thickness (µm)	319.36 ± 90.08	341.60 ± 99.19	318.36 ± 103.01	<0.001
Spherical refraction (D)	-0.49 ± 0.87	-3.52 ± 3.75	0.42 ± 1.26	<0.001
Cylindrical refraction (D)	-0.66 ± 0.62	-1.10 ± 1.15	-0.69 ± 0.63	0.047
Axial length (mm)	23.26 ± 0.83	23.29 ± 0.84	23.27 ± 0.84	0.003

a = One-way ANOVASignificant differences are shown in **bold**. SD= standard deviation.

### Comparison of eye color and effectiveness of eye drops

The analyses performed were compared between blue and brown eyes only because there were an insufficient number of participants with green and hazel eyes. An examination of the change in spherical refraction and pupil diameter for different eye colors revealed a significant difference in these measurements in blue eyes after pilocarpine administration (Table 3).

**Table 3:** Comparison of the effects of eye drops according to eye color

Measurements		Mean ± SD (blue eyes, n=8)				Mean ± SD (brown eyes, n=20)			P^a^ value	
Spherical refraction without drops		-0.38 ± 0.18 D				-0.62 ± 0.39 D			>0.050	
Spherical refraction with Pilosed		-7.87 ± 1.53 D				-2.13 ± 0.50 D			**0.001**	
Spherical refraction with Sikloplejin		0.58 ± 0.32 D				0.25 ± 0.36 D			0.480	
Pupil diameter without drops		3.16 ± 0.15 mm				3.10 ± 0.24 mm			>0.050	
Pupil diameter with Pilosed		1.50 ± 0.09 mm				1.96 ± 0.07 mm			**0.001**	
Pupil diameter with Sikloplejin		5.74 ± 0.25 mm				6.01 ± 0.41 mm			0.230	

a = Mann-Whitney *U* test. SD= standard deviation.

## DISCUSSION

In the present study, we found a significant increase in choroidal thickness and axial length in pharmacologically accommodated eyes, and a significant difference in central macular thickness between accommodated eyes and cycloplegic eyes. Our results showed that pharmacological accommodation and cycloplegia modify not only the lens and anterior chamber of an eye, but also the retina and choroid. Furthermore, ocular measurements may be affected by pharmacological accommodation and cycloplegia. In addition, blue eyes exhibited more pronounced differences in terms of spherical aberration and reduction in pupil diameter after pilocarpine instillation when compared with brown eyes.

The comparison of subfoveal choroidal thickness measurements revealed a significant increase in choroidal thickness as a result of pilocarpine instillation. However, we did not encounter such a difference after cyclopentolate instillation. Mwanza et al. reported no significant difference in the subfoveal choroidal thickness analysis performed after mydriatics administration^(1)^. Conversely, a study of healthy children, performed by Zhang et al., found a significant increase in subfoveal choroidal thickness after cycloplegia, obtained through atropine^(2)^. In their analysis of the effects of different cycloplegic drops on subfoveal choroidal thickness, Yuvaci et al., obtained significant results indicating that mydriasis could reduce subfoveal thickness^(3)^. In another study, Kara et al. observed a decrease in choroidal thickness after using phenylephrine and tropicamide, with increased pupil diameter^(4)^. Consequently, it is impossible to draw clear conclusions about the effect of choroidal thickness on cycloplegia. Regarding accommodation, Woodman et al. observed a significant decrease in choroidal thickness in patients with long-term accommodation^(10)^. In another study, Woodman-Pieterse et al. found a significant decrease in choroidal thickness as a result of 6 D accommodation^(11)^. Our findings showed that pharmacological accommodation resulted in a significant increase in choroidal thickness. The difference between the findings of the present study and those of previous studies could be attributed to procedural differences in obtaining accommodation. As explained above, we obtained accommodation through a pharmacological process, which was different from the other studies. Pilocarpine instillation may result in vasodilation of the choroidal vessel. Therefore, we do not know whether participants underwent accommodation during the measurements. The present study’s results showed that pilocarpine increases choroidal thickness. Since a pilocarpine-induced increase in retinal detachment prevalence has now been assessed, we believe that further investigation is needed to determine whether such intraocular physical changes affect certain conditions, such as retinal detachment. In addition, further studies using larger sampling groups may shed light on this topic.

Here, we found that the axial length was significantly higher in the measurements performed with pilocarpine than in the measurements without drops and with cyclopentolate. Chang et al. found no significant change in the axial length in the volunteers whose eyes were rendered cycloplegic^(7)^. Bhatia found no significant change in the axial length after cycloplegia induced through tropicamide and homatropine^(8)^. In addition, in a study performed by Huang et al., no significant change was found in the axial length after cycloplegia^(6)^. Moreover, Cheung et al. did not detect significant changes in the axial length after cycloplegia^(9)^. Conversely, Cheng et al. detected a significant increase in the axial length after cycloplegia induced using tropicamide^(12)^. Gao et al., distinguished two different groups, i.e., hypermetropic and myopic, and analyzed the effects of cycloplegia on the axial length^(13)^. The axial length increased in hypermetropic eyes but decreased in myopic eyes. Although studies have shown that cycloplegia, typically, does not have a significant effect on the axial length, there are insufficient studies wherein patients are grouped according to early-onset refractive error and measurements are performed. In the present study, we detected a significant increase in the axial length with pharmacological accommodation. Similar to our study, Woodman et al. detected an increased axial length after long-term accommodation^(11)^. In a study of pseudophakic eyes, Shao et al. showed that pilocarpine-induced accommodation elongated the axial length^(14)^. Our study is important, as it clearly associated axial elongation with phar macological accommodation. The various studies revealed that accommodation increased axial eye length, which may contribute to the development of myopia^(15,16)^.

In our analyses, cycloplegic and accommodated eyes showed no significant change in macular thickness compared with the measurements performed without drops; however, there was a significant difference between the measurements of accommodated and cycloplegic eyes. Moreover, the significant differences observed between cycloplegia and accommodation suggest that these two opposite conditions lead to significant changes in macular thickness. There are few studies comparing the effects of the cycloplegic and accommodative conditions on the macular and optic nerve thickness measurements. Despite the significance of the present study’s results, studies using a larger sample size are needed to standardize the tests. In addition, these conditions can lead to different results in diseases affecting macular edema and the optic nerve fiber layer. Further studies carried out not only on healthy volunteers but also on patient groups with such diseases, such as diabetic macular edema, are needed.

We found increased pilocarpine sensitivity in participants with light-colored eyes. These participants were more likely to have side effects, such as pilocarpine-related nasal discharge, watering, and eye pain. We also observed higher myopic shifts and a more pronounced reduction in the pupil diameter when we performed refraction measurements. Our statistical analyses revealed that pupil diameter and myopic shift values were significantly different between blue and brown eyes. Therefore, we think that eye color may affect the pharmacodynamics of other drugs.

In general, the limited number of patients and patient demographic similarities were limitations of the present study. Our study was performed on healthy young volunteers, but most polyclinic patients are elderly or individuals with eye problems. In particular, the manner in which these measurements are affected in ophthalmological conditions, such as cystoid macular edema and cataract, requires a separate, extensive study. Pupil diameter reduction in accommodated eyes may have affected the results, particularly in instruments that are based on the reflection of different light wavelengths from the surface.
